# Analysis of learning curves in predictive modeling using exponential curve fitting with an asymptotic approach

**DOI:** 10.1371/journal.pone.0299811

**Published:** 2024-04-18

**Authors:** Leonardo Silva Vianna, Alexandre Leopoldo Gonçalves, João Artur Souza

**Affiliations:** Graduate Program in Knowledge Engineering, Management, and Media, Federal University of Santa Catarina, Florianópolis, Santa Catarina, Brazil; Cairo University, EGYPT

## Abstract

The existence of large volumes of data has considerably alleviated concerns regarding the availability of sufficient data instances for machine learning experiments. Nevertheless, in certain contexts, addressing limited data availability may demand distinct strategies and efforts. Analyzing COVID-19 predictions at pandemic beginning emerged a question: how much data is needed to make reliable predictions? When does the volume of data provide a better understanding of the disease’s evolution and, in turn, offer reliable forecasts? Given these questions, the objective of this study is to analyze learning curves obtained from predicting the incidence of COVID-19 in Brazilian States using ARIMA models with limited available data. To fulfill the objective, a retrospective exploration of COVID-19 incidence across the Brazilian States was performed. After the data acquisition and modeling, the model errors were assessed by employing a learning curve analysis. The asymptotic exponential curve fitting enabled the evaluation of the errors in different points, reflecting the increased available data over time. For a comprehensive understanding of the results at distinct stages of the time evolution, the average derivative of the curves and the equilibrium points were calculated, aimed to identify the convergence of the ARIMA models to a stable pattern. We observed differences in average derivatives and equilibrium values among the multiple samples. While both metrics ultimately confirmed the convergence to stability, the equilibrium points were more sensitive to changes in the models’ accuracy and provided a better indication of the learning progress. The proposed method for constructing learning curves enabled consistent monitoring of prediction results, providing evidence-based understandings required for informed decision-making.

## Introduction

The ability to build optimal data models using machine learning algorithms has become a critical issue in the era of Big Data. The existence of large volumes of data has considerably alleviated concerns regarding the availability of sufficient data instances for machine learning experiments. Nevertheless, in certain contexts, addressing limited data availability may demand distinct strategies and efforts. In these contexts, understanding how the amount of data affects the reliability of predictions contributes to the overall effectiveness of predictive models and ensures high-quality information for decision-making.

The intense scientific development experienced during the COVID-19 pandemic resulted in positive impacts across diverse domains. Statistical methodologies and machine learning algorithms have played a relevant role in discovering patterns and trends within data, intending to achieve the most accurate models for predicting pandemic behavior. Moments of crisis have, remarkably, propelled innovation and transformation. Since the onset of the COVID-19 pandemic, the world has experienced profound changes that have affected various aspects of life. Environment, Politics, Economics, and terrorism have already been responsible for many disruptive events. At this time, Health has been the focus and the disease caused by the SARS-CoV-2 virus has transformed societies worldwide.

Government bodies and policymakers have executed vital strategies to manage the pandemic, including health resource allocation and sanitary surveillance planning. There is a broad consensus that quality information is crucial for making informed decisions to control the spread of COVID-19 [1–9]. Therefore, data analysis and the development of models that provide sufficient confidence for decision-making are essential. Machine learning algorithms and advanced statistical methods to model non-linear and non-parametric time series can provide the knowledge necessary for pandemic control. The pandemic evolution patterns comprehend the necessary information to predict the future incidence of the disease [5, 7, 10–13]. Time series models have been employed to estimate the size and impact of numerous infectious diseases globally, including but not limited to Zika virus outbreaks in 2016, measles in China and Britain, influenza mortality in France, Ebola virus in Africa, smallpox in Bangladesh, the H1N1 outbreak in 2009, and influenza in Hong Kong [14].

COVID-19 pandemic scenarios have been monitored through the data generated by surveillance services and tracing epidemic curves. Early studies about the epidemic curves of COVID-19 in Brazil were produced considering distinct aspects of the disease. Pereira et al. [5] stated the need for correct planning for resource allocation; Salgotra et al. [15] studied the transmission dynamics of the disease in highly affected countries, including Brazil; and Cotta et al. [2] examined public health interventions in different scenarios. Likewise, da Silva [12] evaluated the interference of exogenous climatic variables in COVID-19 case and death forecasting, and limited data were considered for prediction in the research developed by Hawas [13]. A susceptible-infected-removed model, including a component for patients with no or few symptoms (A-SIR model), was applied by Neves and Guerrero [16] using COVID-19 data from a Brazilian State. These studies used machine learning or advanced statistical techniques for predictive modeling when the amount of data was yet scarce.

Forecasting accuracy can be analyzed with a learning curve generated with the prediction errors in different subsets by incrementing the amount of available data [17–19]. Learning curves are traditionally two-dimensional Cartesian graphs representing a set of points that describe how the performance of a classifier is related to the sample size [20]. They have been used in various fields of knowledge, such as Health, Biology, Chemistry, Social Sciences, Economics, Engineering, Agriculture, and Business, to measure performance evolution over time [10]. They can also be used to determine the minimum sample size for the appropriate performance of machine learning algorithms [18].

Therefore, a key question arose at the start of the pandemic: how much data is needed to make reliable predictions? When does the volume of data provide a better understanding of the disease’s evolution and, in turn, offer reliable forecasts? Given these questions, the objective of this study is to analyze learning curves obtained from forecasting the incidence of COVID-19 in Brazilian States using ARIMA models with limited available data.

The research presented in this article offers significant contributions by investigating the equilibrium point in the temporal evolution of data volume to develop accurate models. By establishing the optimal threshold for the size of a dataset, this research provides a practical approach to enhancing the forecast accuracy of data models. We evaluated the behavior of COVID-19 predictive modeling during the early critical stages of the pandemic when data availability was severely limited. Despite numerous studies on predictive modeling in the scientific literature during this period, none proposed investigating the dynamic changes in model results over time. Applying learning curves to address this issue, our research also sheds light on the future performance of data models used for forecasting tasks.

## Background

### Autoregressive Integrated Moving Average

Autoregressive Integrated Moving Average (ARIMA) is a modeling technique crafted for analyzing and predicting time series data, characterized by observations systematically collected at regular intervals over time. ARIMA models represent an improvement of the autoregressive model (AR), which relies on a linear combination of previous observations, and the moving average model (MA), built upon residual errors from prior predictions. The model also has an integrated component (I) to express the nonstationary behavior of a time series. ARIMA combines these three components (AR, I, and MA) to model and forecast time series data, addressing autoregressive and moving average dependencies while accounting for nonstationary behavior through differencing. The order of the ARIMA model is denoted by the parameters *p*, *d*, and *q*, representing the orders of the autoregressive, integrated, and moving average components, respectively [21].

Setting the parameters (*p*, *d*, and *q*) for an ARIMA model is a required step in processing time series data, demanding careful consideration. Inaccurate parameter selection can lead to poor forecasts. The current research involved building a substantial number of models, and, to address this complexity, automated forecasting was considered.

### Symmetric Mean Absolute Percentage Error

The Symmetric Mean Absolute Percentage Error (sMAPE) stands as a compelling metric to measure the precision of forecasting models. This intuitive measurement is obtained from the average of the absolute percentage discrepancies between predicted and actual values. The essence of sMAPE lies in its ease of interpretation, expressed as a percentage reflecting the proportions of errors relative to actual values.

Various authors have employed sMAPE as a benchmark for model prediction evaluations, albeit with slight alterations, resulting in a range of value variances [22]. For instance, Nazir et al. [23] assessed a model developed to forecast the future energy demand of customers within a smart grid setting. They incorporated a two-multiplying constant in the sMAPE formula, yielding results ranging between zero and two. These results could also be represented as a percentage value. Similarly, Kırbaş et al. [24] utilized a comparable equation to assess models, derived from various methods including ARIMA, to predict COVID-19 trends across numerous countries.

The pioneering equation proposed by Armstrong [25] also featured the two-multiplying constant, leading to a maximum sMAPE value of two, which could be misinterpreted. To address this issue, some authors have eliminated this multiplying constant, resulting in a simpler-to-interpret range between zero and one. For example, Zaghloul et al. [26] adopted this latter equation for evaluation of models simulating the performance of biomass reactors in real time, using different machine learning algorithms.

Considering these aspects, for this research, we chose to employ sMAPE to measure the relative errors between the actual values of a testing dataset and the predicted values. This was achieved using the equation:

$$sMAPE=\frac{1}{n}\sum_{t=1}^{n}\frac{\left|a_{t}-p_{t}\right|}{\left|a_{t}|+|p_{t}\right|}$$ (1)

where *n* is the number of samples, *a*_*t*_ is the actual value and *p*_*t*_ is the predicted value in a certain time *t*.

When conducting various model evaluations, it is crucial to use a relative error measure that simplifies a comparison between multiple models obtained from distinct datasets. Relative metrics–for instance, Mean Absolute Percentage Error (MAPE) and sMAPE–have advantages over absolute metrics—Mean Absolute Error (MAE), Mean Squared Error (MSE), and Root Mean Squared Error (RMSE)—in certain contexts. Relative metrics are not affected by the scale of the values being measured and are intuitively interpretable as percentages. Moreover, although MAPE is a widely utilized relative error metric, it suffers from a known limitation of underestimating the error when predictions are lower than the actual values. Consequently, employing the sMAPE (or adjusted mean absolute percentage error) addresses this bias and ensures a more accurate assessment of model performance [25].

The sMAPE overcomes the drawbacks of MAPE by symmetrically calculating the absolute percentage difference between predicted and actual values, therefore providing a balanced evaluation of model accuracy regardless of whether errors are overpredicted or underpredicted. One disadvantage of sMAPE is that it tends to be susceptible to null values, which can lead to division by zero errors. Nevertheless, by applying sMAPE as a relative error metric, we confidently compared and evaluated the performance of ARIMA models across different datasets in our research.

### Curve fitting

Curve fitting is a mathematical technique that seeks to find the most suitable function to represent a given set of data points, effectively modeling a real-world phenomena, and providing a comprehensive description of their behavior. The underlying objective is to establish a clear relationship between dependent and independent variables. This process involves utilizing various regression techniques structured to minimize the disparity between the mathematical function and the actual data values.

To determine the optimal mathematical function, heuristic approaches are employed, considering the knowledge about the specific phenomenon being modeled. The parameters of the selected function are adjusted through optimization techniques such as the least squares method, gradient method, global optimization algorithms, and nonlinear regression. The ultimate result of this fitting process is a curve that can be effectively used to predict future values and explore the underlying patterns and trends present in the data.

Even the most complex problems can be effectively addressed through curve fitting. By creating, transforming, and developing a robust mathematical model, researchers can tackle challenging issues characterized by complexity, non-linearity, memory effect, or stochastic structures. Such scenarios require the application of specialized modeling and solution methods [27]. Parameter estimation plays a pivotal role in curve fitting to find the best possible fit for the observed data. This critical step ensures that the developed mathematical model yields more realistic values for its parameters, resulting in precise and accurate approximations through the curve fitting process [28].

Furthermore, forecasting data using curve fitting techniques offers an approach for modeling complex problems and obtaining information into the studied phenomena. By adapting the mathematical function to real data, it ensures accurate approximations that can significantly improve forecasting capabilities [29]. Curve fitting defines a correlation between raw data and predictions, allowing for a deeper understanding of relationships within the data, which can be employed to model various experiments, exemplified by its application in scenarios like learning curves.

Learning curves inherently demonstrate a typical behavior. They exhibit a symmetric reduction in errors over time, finally stabilizing or converging to a final plateau [10, 30, 31]. This decrease in errors and convergence to a plateau symbolizes the learning or improvement that occurs with an increase in training or in data volume. Various curve types such as linear, quadratic, cubic, power-law, inverse, exponential, double exponential, logarithmic, logistic, or Weibull can fit such behaviors [10]. Each of these curves possesses unique attributes and is best suited for specific types of data trends.

In this research, we opted for an asymptotic exponential curve to model the dataset of prediction results, obtaining a learning curve as outcomes. This choice was influenced by the characteristics of the sequential sMAPE results, exhibiting a pattern that was appropriately captured by an asymptotic exponential curve. The least squares method was employed to estimate a 3-parameter model by approximating the solution and minimizing the sum of the squares of the residuals, using the function:

y=a∙exp(−b∙x)+c (2)

where *a* is the amplitude, *exp* is the exponential function with the base of the natural logarithm, *b* is the decay constant, *x* is the independent variable, and *c* is the vertical shift of the function *y*.

A negative value for the parameter *b* was adopted to capture the descending trajectory of the exponential curve. This adjustment ensured that the fitted curve accurately represented the decay in prediction errors over time.

### Average derivative

The average derivative is a comprehensive concept in regression analysis, representing the mean slope of a regression curve. This functional measure is derived from the joint distribution of predictor variables and the response variable [32], or it can be described as the mean gradient [33].

The average derivative provides a weight of the sum that offers a straightforward representation of the relative impacts of individual predictor variables on the response variable. By quantifying the average change in the response variable for a unit change in each predictor, the average derivative provides interpretable coefficients that effectively measure the relative impacts of separate predictor variables on the mean response [34]. Finally, the average derivative is a useful method for data summarization, providing information about how predictor variables influence the overall behavior of the response variable.

Within the scope of this research, we employed the average derivative to assess the pattern of the asymptotic exponential curve. By applying this metric, we aimed to represent the observed trend in the data, enabling us to capture the convergence exhibited by the curve, and adequately interpret the results.

### Equilibrium value

An equilibrium value can inherently define the equation curves represented by a hyperbola and its asymptote. Consequently, the method introduced by Ford [35] and Walford [36] can be applied to calculate this equilibrium value (*L*_*∞*_), which is interpreted as the limit value of the *y*_*i*_ tendency while *x*_*i*_ increases or decreases, where *i* is the iteration index.

Let us suppose a data series (*x*_*1*_, *y*_*1*_), (*x*_*2*_, *y*_*2*_),…, (*x*_*i*_, *y*_*i*_) fitted by an equation *y*_*i*_ = ƒ(*x*_*i*_) and, when *x*_*i*_ tends to the infinite (*x→∞*), the expected behavior is *y*_*i*_ ≅ *y*_*i+1*_. And considering the existence of an equilibrium value, it is possible to declare that *L*_*∞*_ ≅ *y*_*i*_ ≅ *y*_*i+1*_.

Hence, the Ford-Walford method defines a function (ƒ):

$$y_{i+1}=f(y_{i})$$ (3)

that fits the (*y*_*i*_, *y*_*i+1*_) pairs.

Applying a linear regression function for a 2-parameter model yields:

$$y_{i+1}=a∙y_{i}+b$$ (4)

where *a* is the scaling factor and *b* is constant term.

And, as *L*_*∞*_ ≅ *y*_*i*_ ≅ *y*_*i+1*_,

$$L_{\infty }=a∙L_{\infty }+b$$

$$L_{\infty }=\frac{b}{\left(1-a\right)}$$ (5)

The Ford-Walford method is a widely employed technique used to estimate the equilibrium value for biological growth curves. This value represents the hypothetical maximum size or number that a population or organism would attain if it continued to grow indefinitely. In this domain, this type of information plays a pivotal role in understanding the growth dynamics of biological processes and provides information into the long-term behavior of the system.

In the context of our research, this method was applied to determine the asymptote value of the exponential curves. By leveraging the Ford-Walford method, we were also able to identify a limit from which the studied ARIMA model could achieve reliable predictions.

## Experiment

### Predictive modelling

With the objective of obtaining comprehensive evaluation metrics for data model predictions, we conducted a retrospective study on the incidence of COVID-19 across the Brazilian States. Following the acquisition and modeling of the dataset, the model errors were assessed by employing a learning curve analysis. The experiment period comprehended approximately a year: from February 25, 2020 (confirmation date of patient zero in Brazil) to February 25, 2021. For our research, codes in Python were developed to perform the forthcoming procedures executed for the COVID-19 data processing.

Data was gathered from a Brazilian Ministry of Health repository created to disseminate information about COVID-19 [37]. According to its website, the repository included a data panel developed with the purpose of being the official communication platform for the epidemic situation of COVID-19 in Brazil, updated with data from the State Secretaries of Health of the Brazilian federative units through the Influenza Epidemiological Surveillance System (SIVEP-Gripe). Among other information, this repository stored data about new and cumulative quantities of cases and deaths, besides recovered and follow-up numbers of cases, separately for each city, state, and cross-country consolidated data. A comma-separated values (CSV) file was downloaded on March 12, 2021, and, to provide understanding of the data characteristics, some graphics were built and presented in the Results section.

During the data preparation phase, the daily incidence of the disease (new cases) was extracted and organized by state, disregarding any extraneous information present in the database. Consequently, a distinct dataset comprising a daily-frequency time series of COVID-19 incidence was generated for each Brazilian State, which was subsequently utilized for individualized data modeling purposes.

**Fig 1:**
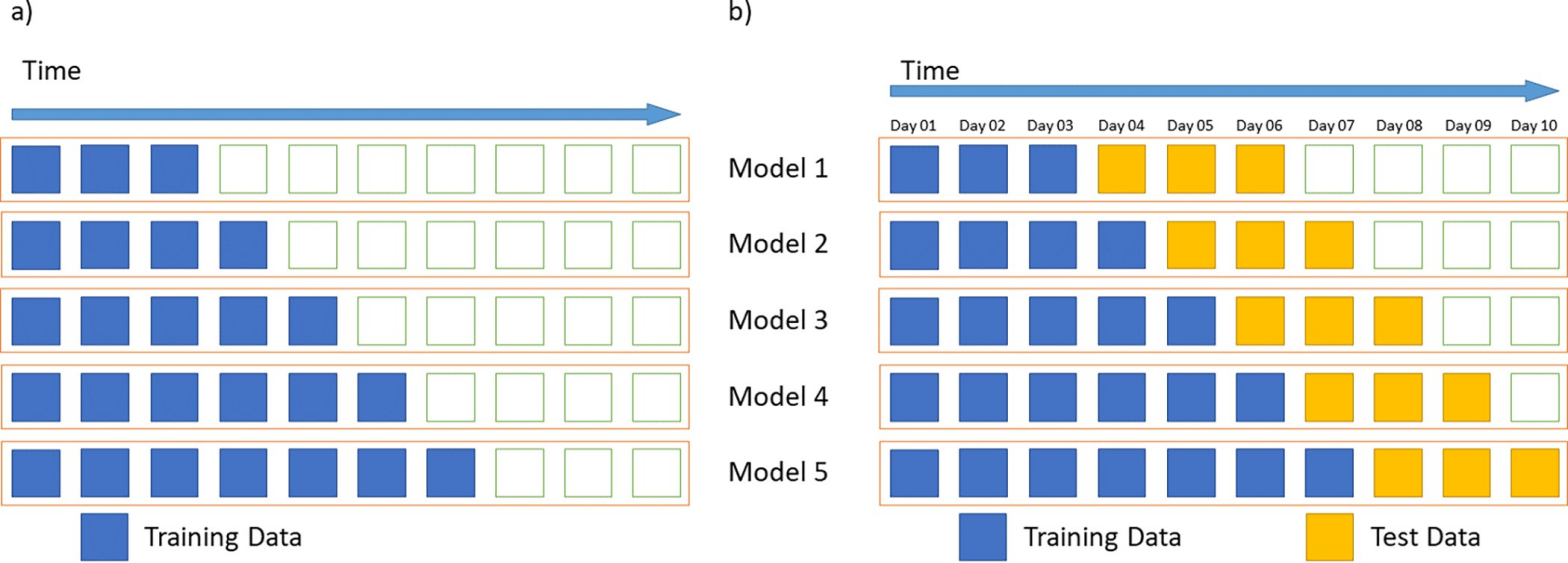
Exemplification of the walk-forward validation used in the experiment. The figure shows a usual walk-forward validation process (a) and the study configuration used for the modeling phase (b). For a better visualization, we used 3-day for initial training and for testing exemplification.

For the modeling phase, an expanding window configuration was executed within a walk-forward validation used for time series data processing (as illustrated in Fig 1), enabling the comparison of the results in different moments of the pandemic evolution. And this study configuration allowed us to measure the model’s suitability for prediction in different sections of the dataset, using the increment of the analyzed data and tracing a learning curve. Each section consisted of at least 14 days of data for model training and precisely 14 days of prediction horizon for testing. While the testing data length was constant, the training data was gradually incremented in one day. The last day of the training data was used as a reference and considered the date of the obtained model.

An ARIMA model was constructed with each section of the training data using a grid search parameter configuration yielded by the *auto_arima* function of the *pmdarima* library. As mentioned in the library documentation, this Python function applies a Canova-Hansen test (for time series with a seasonality component) to determine the parameters of the ARIMA model for the grid search. Using the Akaike Information Criterion (AIC) as a metric, the function finds the ARIMA model with the best parameters and returns it to prediction [38]. The grid search was executed in all sections of the dataset, enabling a different and enhanced parameter configuration for that specific point of the time series, without human interference but considering the data available at that moment.

Likewise, the prediction results were compared with the test data in every time series section. The sMAPE was the evaluation metric used to measure the relative errors between the actual values of the testing dataset and the values predicted by the obtained model. Consequently, an sMAPE error value was acquired at different points of the time series, enabling the creation of learning curves that reflected the increased available data over time.

### Curve analysis

An asymptotic exponential function was chosen to represent the average sMAPE error obtained from the data processing output. Upon analyzing the complete set of average results, we noticed that the models demonstrated an exponential decay in errors, gradually decreasing until they reached a plateau (refer to Fig 6, in Experiment Results). Therefore, we applied the function to effectively fit the sMAPE errors, to analyze their evolution over time. We also conducted other experiment using the Ford-Walford procedure to calculate an equilibrium value of the full set of sMAPE results. The sMAPE and its derivations represented by different graphs characterize the learning curves analyzed in this research.

With the intent to assess the ARIMA models over the time, subsequent sets of the sMAPE results were also processed through an expanding window approach. We exclude ten initial sets from this analysis because they did not exhibit the distinctive pattern suitable for an asymptotic exponential fit. These early sets also contained a limited and less representative number of samples. Nevertheless, they were included before in the curve fit of the complete dataset to ensure a comprehensive analysis of the sMAPE results.

Subsequent curves were collectively plotted together on a graph alongside the sMAPE results, providing an integrated visualization of the curve fitting behavior over time. Additionally, the average derivative was computed for each generated curve as a metric to determine the degree of the asymptotic behavior, or more precisely, their attainment of stability. Following this, the set of average derivatives was plotted to analyze its dynamics and patterns in this time context, directly examining the rate of change over time.

Afterward, we applied subsequent procedures using the Ford-Walford method to calculate an equilibrium value, in the same sets of data. The equilibrium values for each set generated through the expanding window approach were obtained. Consistent with our comprehensive analysis approach, we thoroughly examined the complete dataset to gain an understanding of the overall behavior. We found that the temporal behavior of the equilibrium value exhibited characteristics that could also be represented by an asymptotic exponential function (see Fig 8, in Experiment Results). Consequently, we calculated the derivatives from the curves fitted with this function, intending to analyze its convergence to a stable pattern.

In the last step of our analysis, we conducted a comprehensive comparison of both approaches (average derivative and equilibrium value calculations) by using various samples from the set of sMAPE results, which allowed us to simulate the data available at specific time points. To ensure a robust evaluation, we divided the samples into segments: 25%, 50%, and 75% of the dataset. This comparative analysis aimed to assess the convergence rate and the stabilization of the model’s results. By observing how the models performed at distinct stages of data availability, we could provide information into their learning trajectory and predictive accuracy as more information was incorporated.

Moreover, to further validate our findings, we reproduced this same comparison using the equilibrium value of the full set of sMAPE results as a reference point. This additional analysis allowed us to assess the consistency and reliability of the observed patterns.

## Experiment results

**Fig 2:**
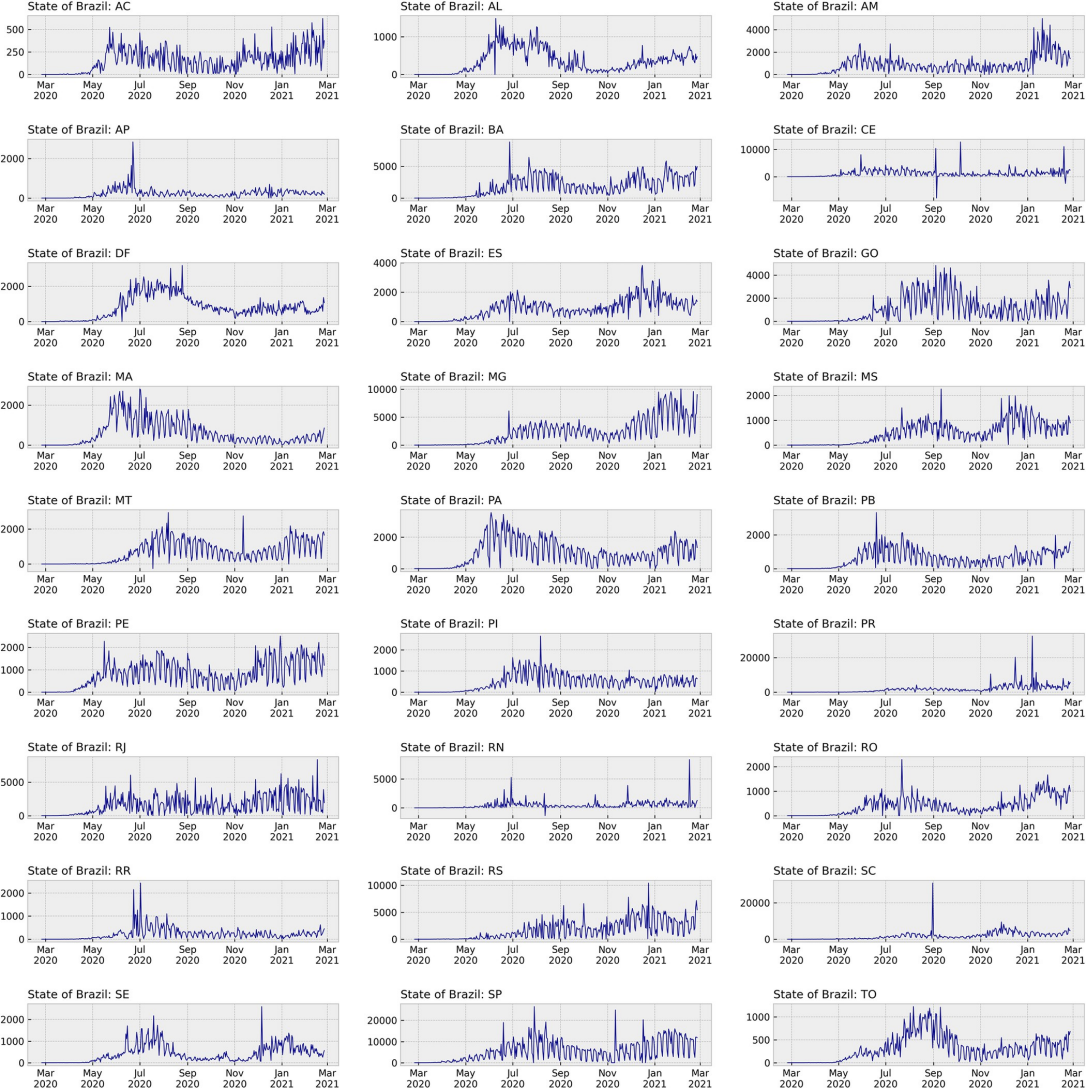
COVID-19 daily incidence in each Brazilian State. The individual graphs exhibit the distinct seasonality behavior of seven days.

**Fig 3:**
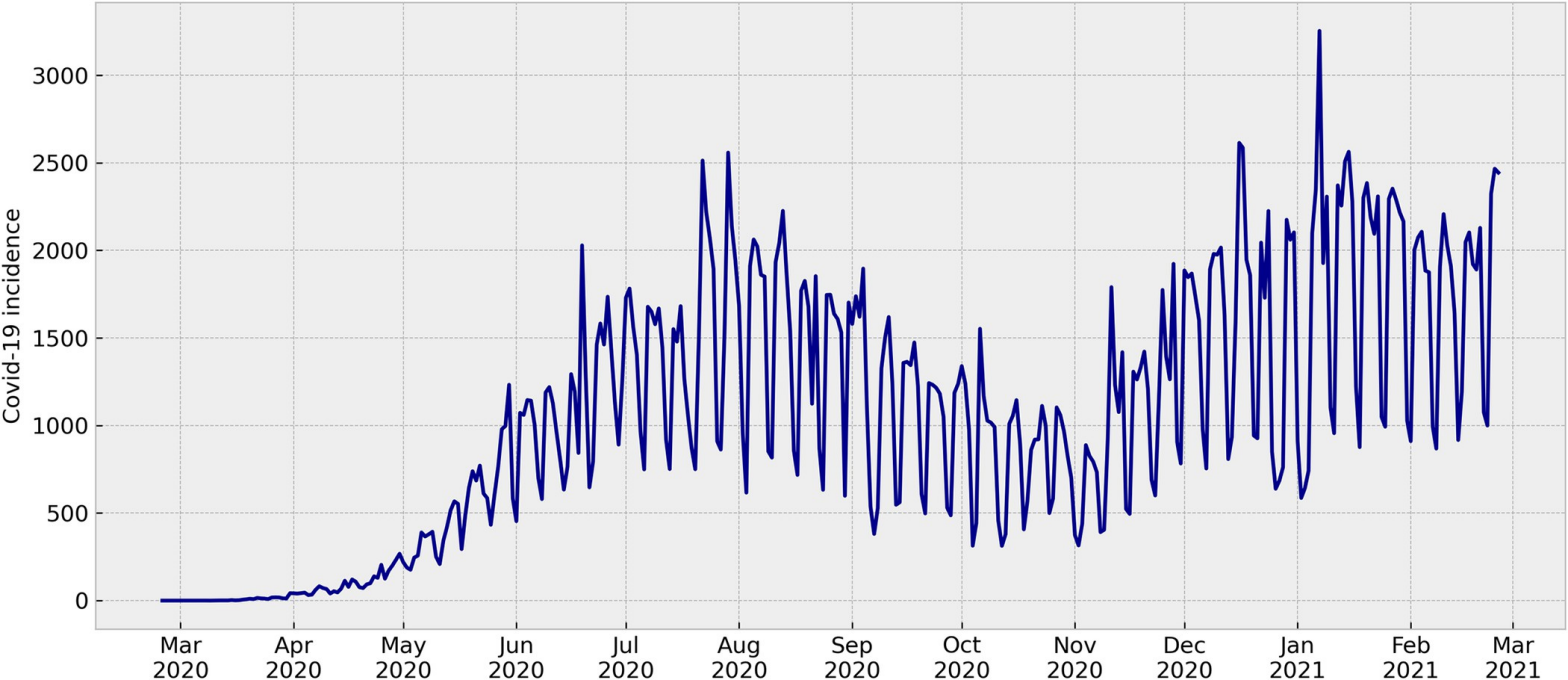
Overall COVID-19 daily incidence in all Brazilian States. The seven-day seasonality behavior is also observed when consolidating all the data in one chart.

After preprocessing the collected data, we generated informative graphics illustrating the incidence of COVID-19 in each of the Brazilian States, as demonstrated in Fig 2. Additionally, the consolidated data was plotted in Fig 3, representing the total disease incidence across all states. To model the data effectively, we identified a crucial component: the seven-day seasonality. This characteristic observed in the data had a significant role in setting the *auto_arima* function.

Seasonality in ARIMA models is an important aspect and we utilized this value as a specific parameter in the *auto_arima* function. The presence of a seven-day seasonality pattern in the incidence charts is a characteristic commonly observed in Brazilian COVID-19 data. This pattern arose primarily due to data input procedures in the SIVEP health information system, resulting in underreporting during weekends. It is essential to clarify that this seasonal behavior is well known as not influenced by the disease itself but rather by the data recording process.

Although we did not assess the seasonality aspects of the Brazilian COVID-19 dataset in our research, we acknowledged its significance and implemented it as a parameter in the *auto_arima* function. This approach ensured that the ARIMA model had appropriately captured the temporal patterns, resulting in a more accurate and reliable analysis of the COVID-19 incidence data in Brazil.

**Fig 4:**
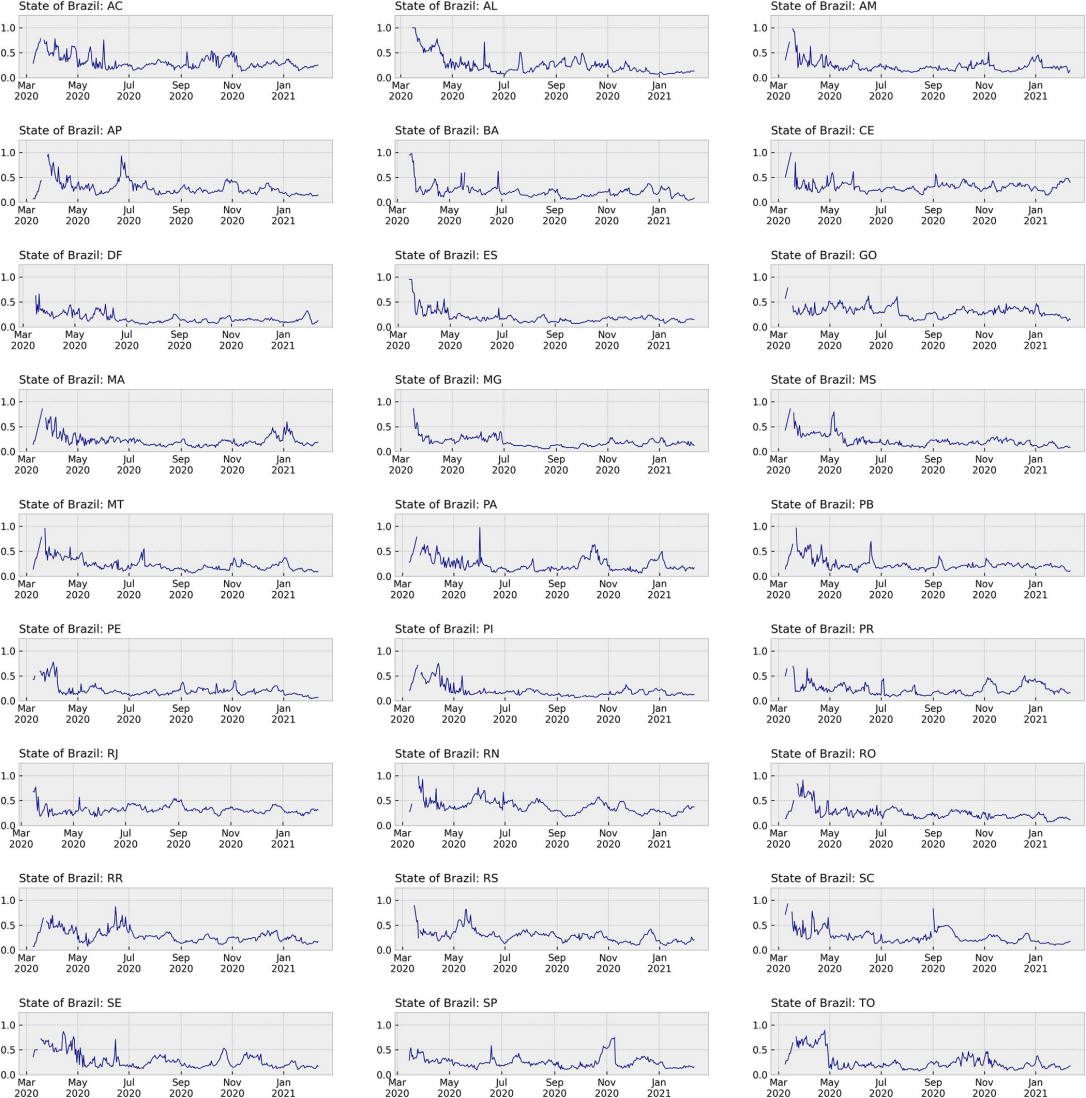
Individual results of COVID-19 prediction for each Brazilian State. The individual sMAPE results are presented by the date of the ARIMA model, with an equal range for comparison purposes.

The sMAPE results of data processing for all reference days throughout the evaluated period in each Brazilian State are presented in Fig 4. To ensure reliable predictions and all steps performed, the model required a minimum amount of training and testing data, leading to the reference period between March 09, 2020 and February 11, 2021. Altogether, 340 distinct models were built for each one of the 27 Brazilian States.

To evaluate the accuracy of the predictions, we employed the sMAPE evaluation metric for calculating the relative difference between the predicted and actual values of COVID-19 daily incidence. Employing this evaluation metric allowed us to assess the results of different Brazilian States without introducing biases, ensuring a comprehensive assessment of the model’s performance. Missing error results were observed as discontinuities in the graphic line when the ARIMA function underwent some inherent calculation errors, which can be observed only in Fig 4.

The results obtained from each Brazilian State demonstrated varying behaviors, which were expected due to the differences observed in the plotted graphics for separate data understanding (Fig 2). While this research did not specifically analyze the causes of these variations, possible factors contributing to the differences could include the spread pattern of COVID-19 or artifacts present in the health information systems used for data recording in distinct states.

**Fig 5:**
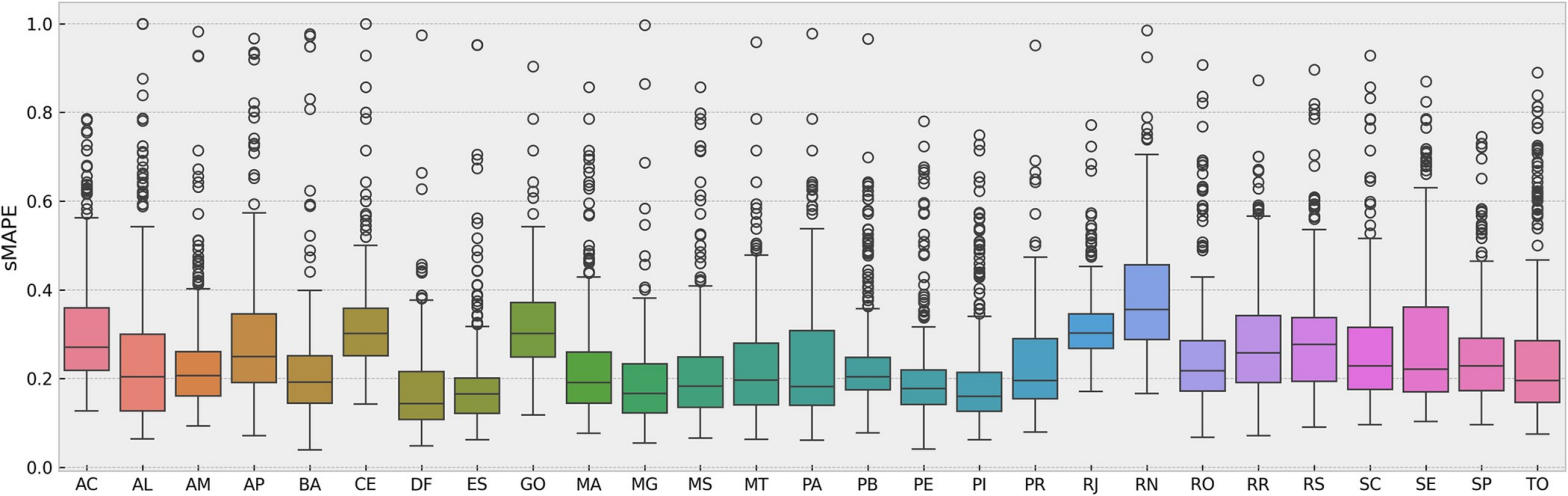
Boxplots of the sMAPE results separately obtained in the Brazilian States. The chart demonstrates the average errors, dispersion pattern, and the occurrence of outliers.

From the boxplots presented in Fig 5, we observed that the Brazilian Federal District (DF) exhibited the lowest average error with an sMAPE of 0.35, while Rio Grande do Norte (RN) had the highest average error at 0.76. Additionally, analyzing the standard deviation of sMAPE values revealed that Rio de Janeiro (RJ) had the lowest dispersion (0.17), whereas Alagoas (AL) had the highest dispersion (0.37). This information highlights the varying performance of the models in different states, indicating potential differences in data quality or patterns of COVID-19 incidence. Despite the variations in individual states, the boxplots show slight differences between most of the states. The presence of outliers was related to higher errors, particularly in the early models with smaller datasets (initial sections in each state of Fig 4). This suggests that the predictive performance of the models improved as more data became available over time.

Table 1 provides a statistical summary, including the number of sMAPE results obtained for each state, which might be unequal due to the calculation errors during the execution of the *auto_arima* function. These errors were not manually corrected to avoid introducing biases in the results through human interference in ARIMA parameter selection. However, when the sMAPE errors were combined by averaging them across all states, the overall learning curve that was produced demonstrated the influence of increasing the amount of available data.

**Table 1:** Statistical summary of the sMAPE results in the Brazilian States. The number of results in each state varies due to calculation errors with the ARIMA function.

Brazilian State	Count	Mean	Standard Deviation	Minimum Value	Maximum Value
AC	337	0.31	0.13	0.13	0.79
AL	335	0.25	0.19	0.06	1.00
AM	337	0.23	0.12	0.09	0.98
AP	335	0.29	0.15	0.07	0.97
BA	333	0.21	0.13	0.04	0.98
CE	337	0.32	0.11	0.14	1.00
DF	334	0.17	0.10	0.05	0.97
ES	335	0.19	0.13	0.06	0.95
GO	336	0.31	0.11	0.12	0.90
MA	337	0.23	0.13	0.08	0.86
MG	334	0.19	0.11	0.05	1.00
MS	337	0.22	0.13	0.07	0.86
MT	337	0.23	0.12	0.06	0.96
PA	337	0.24	0.14	0.06	0.98
PB	336	0.23	0.11	0.08	0.97
PE	335	0.21	0.12	0.04	0.78
PI	336	0.20	0.12	0.06	0.75
PR	335	0.23	0.12	0.08	0.95
RJ	333	0.32	0.08	0.17	0.77
RN	333	0.38	0.13	0.17	0.98
RO	337	0.25	0.13	0.07	0.91
RR	337	0.28	0.13	0.07	0.87
RS	334	0.29	0.13	0.09	0.90
SC	335	0.27	0.14	0.10	0.93
SE	337	0.29	0.16	0.10	0.87
SP	334	0.25	0.11	0.10	0.75
TO	337	0.26	0.17	0.08	0.89

**Fig 6:**
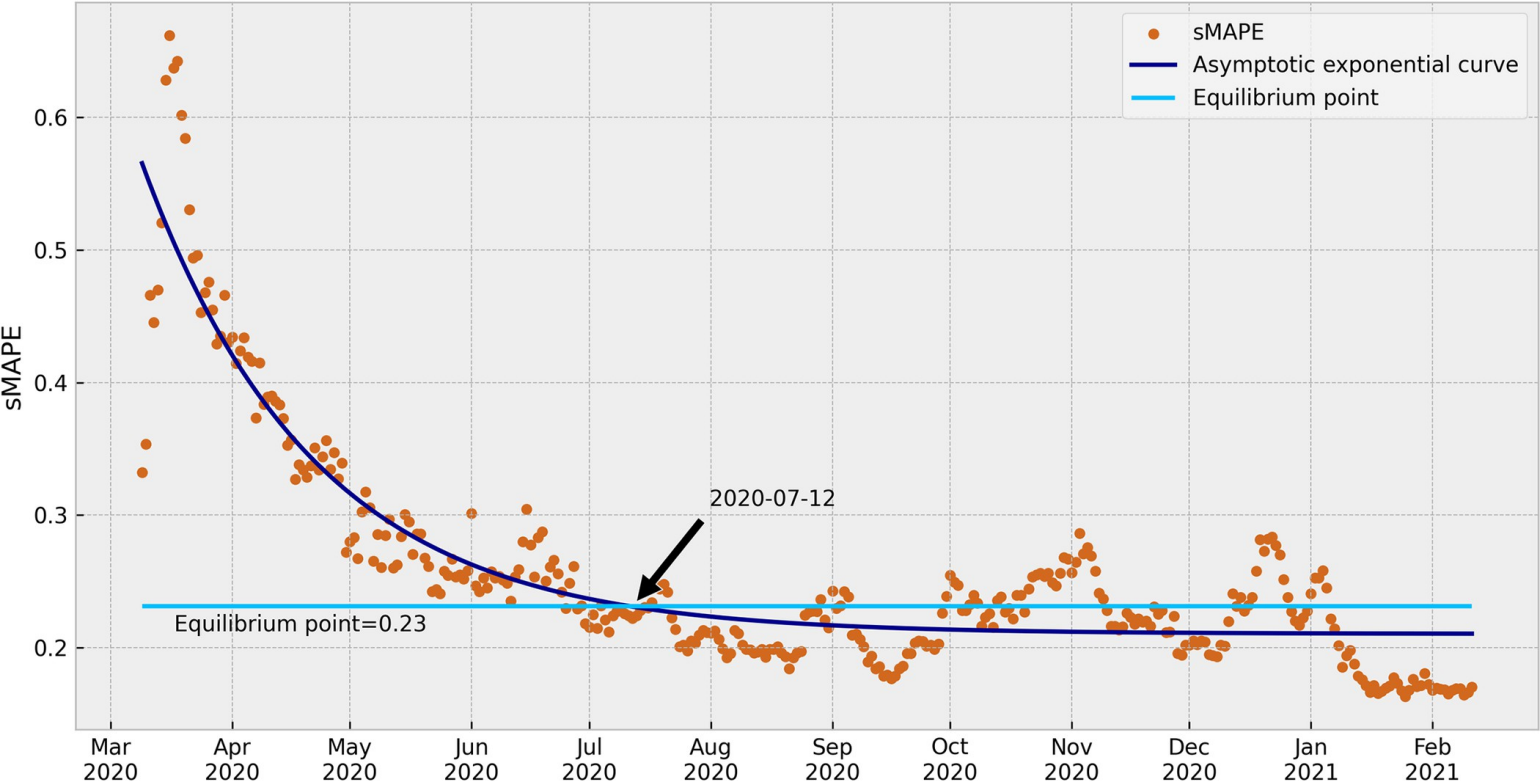
Average of the sMAPE results in all Brazilian States. The graph presents the asymptotic exponential curve fitted, the equilibrium value line (asymptote), and the marked stability point by date of the ARIMA model.

Fig 6 demonstrates the sMAPE results of a 14-day prediction obtained from an ARIMA model, where the average results of all Brazilian States were considered. These aggregated results provide a clearer understanding of the learning curve behavior. There was a noticeable decreasing tendency in the errors of each model as more data was continuously acquired and used for modeling. This finding indicates that the predictive performance of the models improved with the incorporation of additional data, signifying their ability to learn and adapt to the evolving COVID-19 incidence patterns. The learning curve demonstrated a progressive reduction in errors until a certain point, when an average stability was reached. Beyond this point, the errors continued to show variation, but their average values remained relatively constant.

In this study, the convergence to the stability of the ARIMA model results was highlighted by applying an asymptotic exponential function, and an equilibrium point was determined using the Ford-Walford method. The calculated equilibrium point for the full set of results had an sMAPE value of 0.23, which was defined in Fig 6. Based on the curve fitted with the average sMAPE results of all Brazilian States, the ARIMA models were observed to reach the stability point on July 12, 2020. At this stage, this signifies that the ARIMA models had achieved a stable level of performance and were providing reliable predictions of COVID-19 incidence from this point onwards.

**Fig 7:**
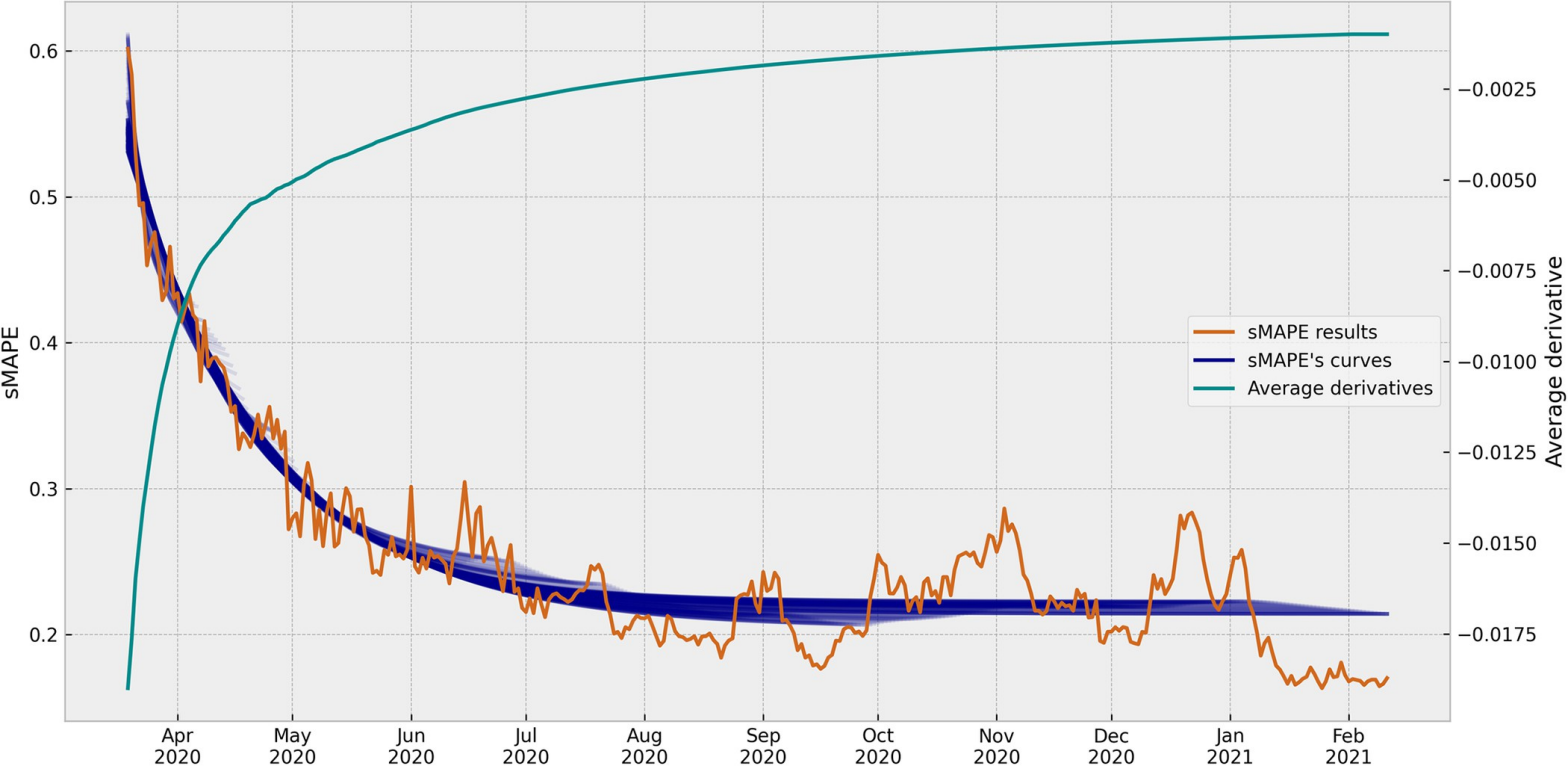
Successive curves of the sMAPE results and their average derivatives. The graph presents the successive asymptotic exponential curves fitted in the set of data and results available on each day of the analyzed period, and, in a different scale, the evolution of the average derivatives calculated for each curve, by the date (day) of the ARIMA model. Although the sMAPE results and the average derivatives were a set of points, they were represented by plotted lines for better visualization.

In the next experiment, we adopted a successive curve-fitting approach using an expanding window to analyze the sMAPE results. Fig 7 presents the set of data and results available on each day of the period. This approach simulated the evolving nature of data and results that a decision-maker would encounter at different points in time.

By plotting all curves together, we observed similar asymptotic behavior in each one of the curves. This finding suggests that as the dataset expanded and added information became available over time, the predictive accuracy of the ARIMA models improved, tending to stabilize the sMAPE values. Fig 7 also represents the evolution of the sMAPE results alongside the successive fitted curves. In this experiment, 330 results from March 19, 2020 to February 11, 2021 were utilized, excluding the initial ten models, considered outliers. Because the ARIMA models rely on the underlying patterns of the time series data, the initial models built on a few instances of the time series failed to capture the pattern structure. Although these outliers were disregarded in the successive curve analysis experiment, they can be seen in the initial section of Fig 6, where is possible to heuristically notice the large error with the asymptotic exponential curve, observing the distance between the initial points and the fitted curve.

To further analyze the convergence of the successive curves, we calculated the average derivative from each curve. The average derivative demonstrated the tendency of the successive curves to approach a plateau or stability over time. This characteristic corroborates that as the ARIMA models incorporated more data and updates, they became more consistent in their predictive performance.

The plotted average derivatives in Fig 7 also provide visual evidence of the evolving stability of the models. As time progressed, the average derivative demonstrated that the different curves evolved towards a more stable behavior. The set of average derivatives displayed an asymptotic exponential behavior, even though it was not explicitly curve-fitted.

**Fig 8:**
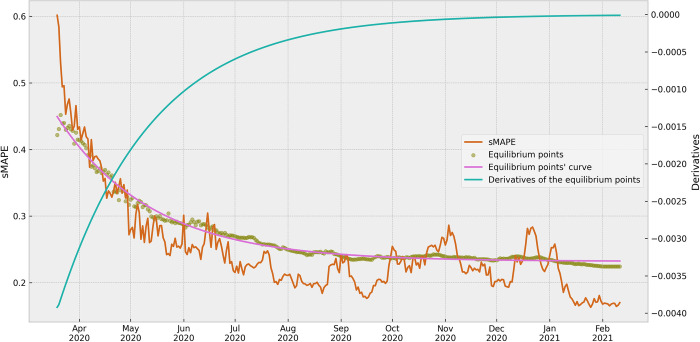
Equilibrium points of the sMAPE results and their derivatives. The graph presents the equilibrium points calculated by Ford-Walford method, the equilibrium point’s curve fitted with an exponential function, and, in a different scale, the successive derivatives of the curve fitted, by the date (day) of the ARIMA model. Although the sMAPE results and the derivatives were a set of points, they were represented by plotted lines for better visualization.

In addition to the average derivative of the sMAPE curves, the successive equilibrium points were calculated from each set of sMAPE results. The equilibrium values were then plotted to ensure the understanding of the temporal behavior of this metric, providing information about the stability and convergence patterns. Fig 8 was used to illustrate the evolutionary behavior of these equilibrium points, along with the curve fitted using the same asymptotic exponential function as for the sMAPE results. By plotting the equilibrium points together with the sMAPE curve, Fig 8 provides a comprehensive view of the model’s performance over time. This graph allowed a direct comparison between the stability of the equilibrium points and the sMAPE values.

The derivatives calculated from the one-curve fitted and shown in Fig 8 also provides valuable information into the model’s behavior. Here, we did not apply the average calculation. These derivatives allowed us to notice and analyze the patterns obtained from the ARIMA models. The convergence of the derivatives to a plateau confirmed the stability of the models, corroborating the findings from the evaluation of the curves.

**Fig 9:**
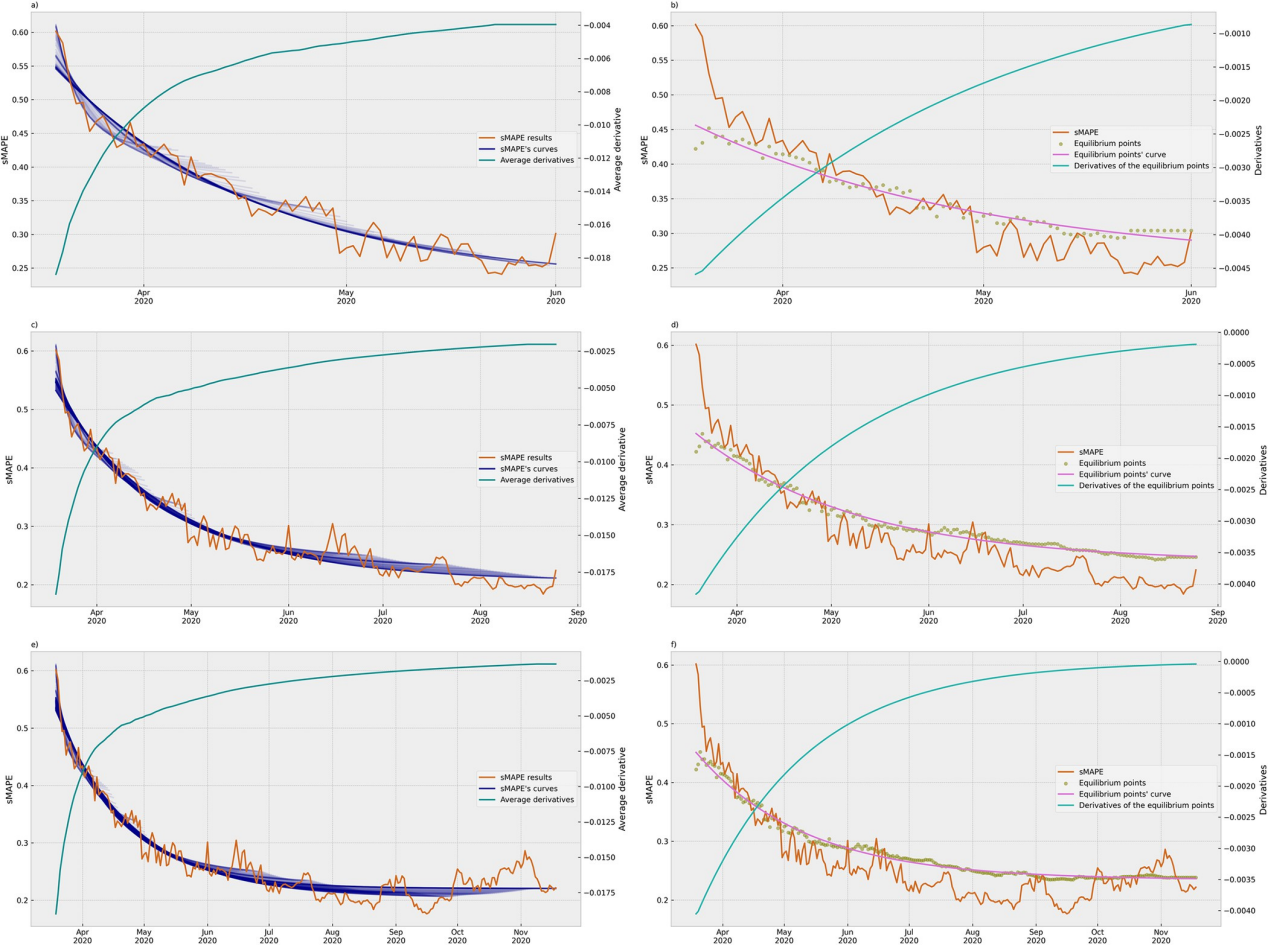
Comparison between the evolution of the average derivatives and the equilibrium points. The average derivatives (graphs a, c, and e) and equilibrium points (graphs b, d, and f) were plotted with 25% (graphs a and b), 50% (graphs c and d), and 75% (graphs e and f) of the sMAPE results, by the date (day) of the ARIMA model. Although the values are represented with different values, the sMAPE results in all graphs are at the same scale allowing the comparison.

In our final analysis, we aimed to attain a comprehensive understanding of the experiment results at distinct stages of the time evolution. We plotted subsequent graphs featuring 25%, 50%, and 75% of the sMAPE results, calculating the average derivative of the curves and the equilibrium points as presented in Fig 9. The subsequent graphs played a crucial role in identifying the convergence of the ARIMA models to a stable pattern. By assessing the accuracy of the models at various intervals, we could compare their predictive capabilities at distinct stages of the learning process.

In the initial graph, which represents 25% of the results, the average derivative displayed an exponential growth that level off, indicating that the convergence to a stable pattern was already reached before. It is important to emphasize that this pattern was not obtained through curve-fitting, but rather as a progression of successive values; however, the plot was represented by a line to enhance visualization. Subsequent graphs of the average derivatives demonstrated the continuation of this convergence pattern, exhibiting a slight growth trend with the inclusion of more data.

In contrast, the successive graphs of the derivatives of the equilibrium values provided a clear and identifiable beginning of the convergence pattern. The first two graphs, representing 25% and 50% of the results, do not show the convergence toward a plateau at that moment. Only in the third one, comprising 75% of the results, a well-defined convergence pattern of the equilibrium values became evident, indicating that the subsequent models had attained a stable level of predictive accuracy.

**Fig 10:**
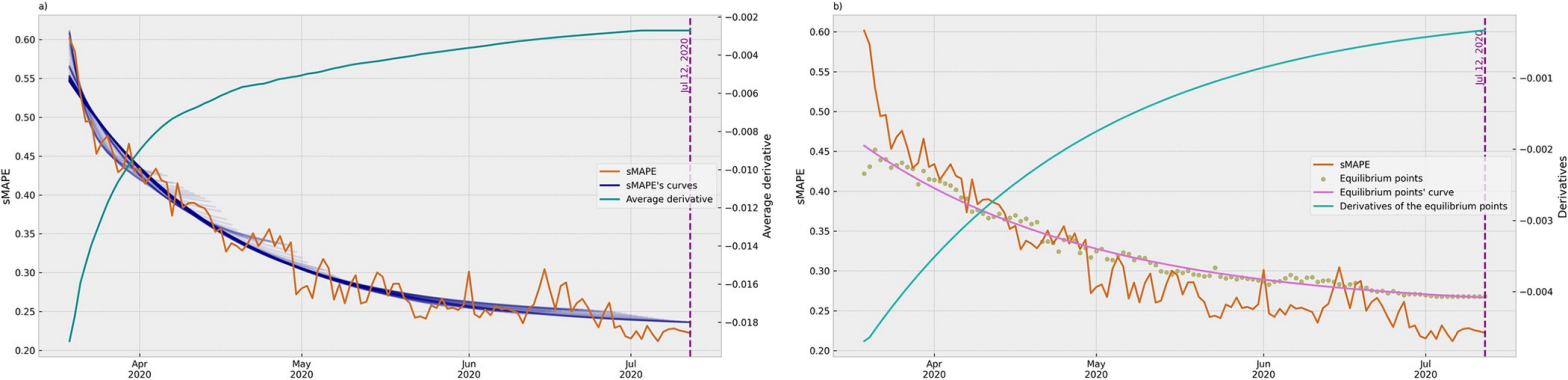
Comparison between the evolution of the average derivatives (a) and equilibrium points (b) at the stability point. The stability point was calculated on July 12, 2020 and the graph is presented by the date (day) of the ARIMA model. The vertical dashed line corresponds to the day of the stability point. Although the values are represented with different values, the sMAPE results in all graphs are at the same scale allowing the comparison.

When comparing both types of graphs using the stability point on July 12, 2020, as the reference, we observed that the equilibrium points began displaying a plateau pattern, indicating a stable state of performance, as shown in Fig 10. In contrast, the graph of the set of average derivatives had already exhibited a convergence pattern before the stability point. This earlier convergence pattern indicated that the average derivative had been signaling the trajectory of models toward stability at an earlier stage. The declining trend in the average derivatives demonstrated a steady improvement in predictive accuracy over time.

## Discussion

Box and Jenkins introduced the Autoregressive Integrated Moving Average (ARIMA) method for time series modeling and forecasting in the 1970s [21]. They highlighted that ARIMA models offer intuitive applicability and are particularly well-suited for modeling various real-world time series, even those with nonstationary behavior. Notably, ARIMA is extensively utilized for time series modeling and effectively handles data with trend, seasonality, and cyclicity [1]. The proficiency of this technique in detecting prevalent patterns within data sequences, inherent to time series analysis, is due to its ability to account for dependencies between successive observations [7]. Considering these attributes, our choice to employ the ARIMA algorithm for COVID-19 prediction was grounded by its proven reliability across diverse datasets and scenarios.

ARIMA models are constructed using only historical data from a time series, and they do not involve recursive learning across different folds of the dataset. This characteristic led us to adopt the study configuration depicted in Fig 2 for our research. The key rationale behind this choice was that the model construction procedure remained independent of the testing (or validation) data subset. This approach ensured that the models assessed at each time step of the series closely resembled an authentic contextual configuration, resulting in unbiased evaluations.

A critical facet of ARIMA involves determining the values for its *p*, *d*, and *q* parameters, which respectively stand for autoregressive, differences, and moving average parameters. Typically, these parameters are ascertained through heuristic analysis of autocorrelation plots [1, 6, 7, 10, 39]. However, considering the substantial number of models built for each Brazilian State, manually configuring these parameters for each time series subset would be infeasible or, at the very least, overly exhaustive, and potentially biased. Consequently, we opted for the automatic configuration of ARIMA parameters, an inherent feature of the *auto_arima* function. The parameter configuration process was conducted without any human intervention, simulating an expected behavior at different moments of the dataset analysis.

Moreover, we decided for a 14-day prediction horizon to ensure the study’s practical relevance in real-world health decision-making scenarios. This choice was made based on the premise that sufficient time is needed for the implementation of health interventions, intending to control the epidemic evolution [2]. Considering disease control into epidemiological weeks, when the decision is taken, it must be implemented in the following week, causing effect only in the next one. Consequently, we executed 2-week predictions to provide sufficient information for decision-making, like Kırbaş et al. [24] and Fong et al. [40], although many researchers have applied a shorter horizon of predictions [1, 6, 12, 15, 39].

Incorporating exogenous data, such as climate and demographic information, has the potential to significantly enhance the accuracy of ARIMA models. A study conducted by Chatterjee et al. [11] demonstrated that COVID-19 incidence is intricately linked to population and population density, with a close interplay between social isolation and disease transmission. Similarly, da Silva et al. [12] highlighted the influence of climate data on COVID-19 predictions, particularly in the context of Brazil, where significant temperature variations had an important impact. Notably, Takele [7] emphasized the importance of temperature and humidity as critical climatic factors for dependable COVID-19 predictions.

It is important to note that the ARIMA function employed in our research can incorporate exogenous variables for time series modeling. One limitation of our study, however, is that we did not utilize exogenous variables, such as demographic, weather, or pollution data. These variables are closely intertwined with the dynamics of COVID-19 spread, and their incorporation, tailored to each Brazilian State, could potentially elevate the quality of our predictions [5]. Multiple variables encompassing environmental, social, political, technological, and economic domains influence the dynamics of COVID-19 incidence [4]. Integrating these variables into data modeling efforts could be explored in future studies.

Considering the adopted evaluation metric, sMAPE enabled a comparison between various models derived from different datasets, measuring the evolution of data quantity over time. Moreover, sMAPE ensured a uniform range of errors across the Brazilian States, thus establishing a coherent and standardized way to assess the predictive capability of different models. By aggregating the sMAPE errors through averaging across all states, the resulting learning curves demonstrated the impact of increasing data availability on model performance.

Learning curves can provide information about the potential enhancement of prediction accuracy through additional data samples. They address questions about the influence of more training data on the performance of modeling and the feasibility of predicting performance extrapolation with increased training samples. Mukherjee et al. [18] employed an inverse power-law fitting to establish the minimum number of samples required for significant performance improvement in genetic microarray cancer classification. Although their concept of learning curve behavior differed from our study, they successfully demonstrated that performance extrapolation could be accurately predicted, assuming a learnable function.

In contrast, Ramsay et al. [10] emphasized the importance of asymptote estimation in evaluating learning curves, representing the final performance level. This aspect is particularly pertinent for health technology assessment, facilitating the measurement of changes in processing performance over time. Their perspective aligns with our approach, which underscores the value of fitting a function to learning curve data. The utilization of learning curves as a tool for performance prediction and assessment holds potential for various fields. By exploring the dynamics of learning curves, researchers can evaluate how additional data samples influence predictive accuracy.

The learning curve exhibited a progressive reduction in errors until it reached a distinct point of average stability. Beyond this inflection point, while the errors continued to demonstrate some fluctuations, their average values remained relatively constant. This observation suggests that the models had reached a level of predictive accuracy stability, implying that further increments in data should not yield substantial improvements in average predictive performance. Consequently, although individual predictions could exhibit some variability due to inherent data complexities, the overall model performance maintains its stability and reliability. When selecting the appropriate curve for fitting, as the asymptotic exponential curve, we could assess the saturation and convergence inherent to the model behavior in our research. Furthermore, our method enabled us to observe and interpret the evolving trends in error reduction.

The progression of average derivatives allowed us to assess the incremental enhancement in predictive accuracy as the models assimilated additional data and updates. The set of average derivatives manifested an intrinsic asymptotic exponential behavior. This pattern signifies the gradual convergence of the average derivative toward a plateau or stable pattern. It could suggest that beyond a certain moment, the potential gains from augmenting the dataset may not significantly enhance predictive accuracy.

Likewise, the behavior of the equilibrium points, with an observed asymptotic tendency reinforced the hypothesis of model stability. The curve fitted in the equilibrium points converged toward a plateau, marking the attainment of stability over a specific timeframe. However, the discrepancy in the timing of convergence patterns between the average derivatives and equilibrium points underscores the distinct nature of these metrics used to assess the models’ performance. The comparison of both types of graphs also highlighted the importance of using multiple metrics to analyze the behavior of data models.

Concerns regarding data sample sizes have historically raised questions about the accuracy of data models [18, 20, 41]. However, in certain critical contexts, determining the required data volume for developing models with robust predictive capabilities is a significant issue. As exemplified during the initial phase of the COVID-19 pandemic, where the amount of data was insufficient to yield reliable predictions [9, 40], discerning the data thresholds for constructing trustworthy models assumes significant importance. In the Artificial Intelligence domain, contemporary contexts also demand an analysis of how sample sizes can impact the outcomes of data models. For instance, Schuurmans et al. [42] researched the prediction of optimal maneuvers for autonomous vehicles on highways within the shortest time possible, investigating the impact of small sample sizes on the reliability of data models.

Exploring other dimensions of Artificial Intelligence, Big Data is characterized by the continuous influx of data, with substantial volume and rapid flow, demanding real-time information extraction. Contexts with a large volume of data seemingly exempt the discussion about small sample sizes. Nevertheless, investigating asymptotic convergence of data models’ outcomes can provide an understanding of how learning performance correlates with the sizes of samples [43], which tend to increase over time. Considering the substantial computational resources invested in training large language models (another current state-of-the-art context in 2023), analyzing the asymptotic behavior of learning curves can anticipate the behavior of data models before extensive training efforts. Consequently, monitoring a learning curve supports the decision regarding the need to retrain a large language model, induced by its temporal quality decay, thereby preventing the expenditure of substantial resources.

The key question driving our research arose from the context of the pandemic: how much data is requisite for reliable predictions? As we evaluated the outcomes obtained from our developed method for learning curve analysis, we assumed its applicability in monitoring large language models. Therefore, we considered another inquiry: at what point does retraining large language models provide substantial performance improvements, factoring in the considerable resources invested in the process? We assumed that the proposed method for learning curve analysis could be aptly suited for this task, founded on the experiment results demonstrating its capability of analyzing the expected future behavior of the models. Consequently, unraveling the answer to this subsequent question remains a prospect for future investigations.

## Conclusions

The trajectory of data volume evolution significantly influences predictive modeling within machine learning algorithms. The data augmentation over time expands the capability of models, resulting in enhanced precision and reliability predictions. Additionally, a larger data volume strengthens model stability by minimizing the influence of anomalies and outliers. This comprehension of data model dynamics affords the ability to anticipate potential risks inherent in adopting predictions, therefore facilitating the mitigation of potential adverse impacts.

Decision-making in dynamic and complex situations, such as the COVID-19 pandemic, demands continuous monitoring of the evolving situation. The initial phases of pandemics highlight the critical significance of relying on evidence-based information within rapidly changing contexts, using real-time data to inform responses. Employing the proposed method for constructing learning curves enabled consistent monitoring of prediction results, providing evidence-based understandings required for informed decision-making.

The application of an asymptotic exponential curve for fitting a set of model results allowed the analysis of learning curves derived from ARIMA models at varying points, mirroring the augmented data availability across time. By evaluating distinct subsets of sMAPE results, we obtained a comprehensive understanding of the model’s evolution and performance at distinct stages. This exploration enabled us to make substantial inferences about the model’s trajectory and its suitability for the designated task. Such understandings demonstrate valuable for decision-making and the refinement of model parameters and strategies.

We observed differences in average derivatives and equilibrium values among the multiple samples, enlightening the capability of these metrics to assess the models’ progression toward stability over time. While both metrics ultimately confirmed the convergence to stability, the equilibrium points were more sensitive to changes in the models’ accuracy and provided a better indication of the learning progress.

Using ARIMA models with exogenous variables can introduce additional information to the model. This inclusion may enhance the model’s predictive power by capturing relationships between the time series and external factors. Additionally, exogenous variables allow the model to address seasonality or other patterns that are not adequately captured by the model’s autoregressive and moving average components. Considering these characteristics, exogenous data availability could also impact model reliability and was a limitation of our research. This impact should be the subject of future studies, allowing a comparison with this research. Likewise, future research could apply the proposed method of analysis of learning curves in other contexts for predicting the behavior of the model’s results, for instance, in monitoring large language models.
